# Exploring the Nature and Challenges Among Physicians in Saudi Arabia Responding to an Inflight Medical Emergency: A Cross-Sectional Survey

**DOI:** 10.7759/cureus.76420

**Published:** 2024-12-26

**Authors:** Adil Khalil Hussien, Saif Ali Aljaghwani, Ibrahim Mohammed Albarrak, Abdullah Rodaini Alanazi, Marwan Mohammed Althubaiti, Faisal Abdullah Alqahtani, Ibrahim Abdullah Alharthi, Hussain Abdullah Alqahtani, Khalid Saeed Alshalawi, Mohammed Faris Alanazi

**Affiliations:** 1 Basic Medical Science Department, College of Medicine, Dar Al Uloom University, Riyadh, SAU; 2 College of Medicine, Dar Al Uloom University, Riyadh, SAU

**Keywords:** air emergency, airline, inflight medical emergencies, legal concerns, life support training, physician preparedness, volunteer

## Abstract

Background

Inflight medical emergencies (IMEs) present a challenging situation due to the availability of limited medical resources and a complex cabin environment. The physicians have an ethical responsibility to aid in such situations. This study aims to assess the attitudes of Saudi physicians regarding IMEs.

Methods

A cross-sectional survey was conducted among medical interns and postgraduate physicians in Saudi Arabia. Data were collected via a structured online questionnaire distributed through professional networks and social media. The survey evaluated physicians’ confidence, preparedness, and barriers to volunteering in an IME.

Results

A total of 368 respondents were included in the study, with a plurality (42.4%, n = 156) in the 30-39 years age group. The proportion of males was 55.2% (n = 203). Among participants, 67.1% (n = 247) had encountered an IME, and 62% (n = 228) had assisted in such a situation. Regarding the nature of IME, 17.9% (n = 66) of the respondents reported respiratory emergency (e.g., bronchospasm). Participants with prior medical emergency experience showed significantly higher scores than those without (54.64 ± 7.81 vs. 49.30 ± 7.10, p < 0.001). Younger physicians (<30 years) displayed the highest confidence scores (54.85 ± 7.03), while those ≥60 years reported the lowest (46.50 ± 4.94, p < 0.01). Physicians with less experience (less than five years) had higher scores compared to their more experienced counterparts (54.69 ± 7.16 vs. 52.25 ± 5.75, p = 0.039). Barriers to volunteering included lack of training (58.6%, n = 215) and legal concerns (61.1%, n = 227), while 67.6% (n = 249) highlighted the need for additional training as a potential motivator.

Conclusion

Overall, the majority of the participants included in our study were willing to aid in an IME. Furthermore, being younger, less experienced, and having prior emergency experience were significant factors in determining the decision to aid in an IME.

## Introduction

Inflight medical emergencies (IMEs) are significant challenges due to the availability of limited medical resources and complex cabin environments. An IME can be defined as a medical event that cannot be handled by the flight crew and requires the assistance of a healthcare specialist [1]. The number of passengers using commercial airlines has increased tremendously in recent years, with approximately 4.5 billion people traveling by air each year [2]. Due to the overwhelming number of people using commercial airlines, medical-related emergencies are, therefore, pretty common. In the United States, one in every 604 flights will have a medical emergency, with a total of 144 emergencies per day in 2019 [3]. In such cases, facing a medical emergency becomes quite common, especially for health professionals who travel frequently [4]. Commercial aircraft fly at high altitudes, but passenger cabins are pressurized to mimic conditions at 5,000-8,000 feet for a safer and more comfortable environment [5]. However, this pressurization can cause gases in the body, like those in the sinuses or middle ear, to expand by around 30%, which might lead to discomfort. People with sinus infections, ear issues, or recent surgeries involving gas pockets are especially prone to experiencing problems during flights [6].

At high altitudes, the aircraft cabin has lower oxygen levels, causing mild hypoxia in healthy passengers, with oxygen saturation dropping from 97% to 93% [7]. This effect can be worse for passengers with lung conditions, who may need extra oxygen or adjustments to their existing oxygen flow during the flight. Long periods of sitting and reduced oxygen can slow venous blood flow, spark inflammation, and activate platelets, which may increase the risk of blood clots during flights [8]. While the exact risk compared to the general population is debated, deep vein thrombosis or pulmonary embolism symptoms often appear hours to days after travel but can also happen during or after long or multiple flights. High-risk passengers may face up to a 5% risk of clots, with symptomless clots occurring in as many as 10% of long-haul travelers [6]. Most medical emergencies are handled by the flight cabin crew, but when the medical event proves dangerous or difficult, medical assistance will be requested from the passengers, upon which medical personnel should identify themselves and participate in providing health care [9].

Although medical assistance is requested in such an event, physicians may be hesitant to respond because of the legal and financial implications in case the victim or his family files a lawsuit, especially since he or she might not be covered by the malpractice policy or because of the stress from the responsibility in such an uncomfortable situation [10]. Furthermore, many healthcare practitioners are hesitant to volunteer in emergency situations because of fear that they might harm the victim by ill-preparedness, even with good intentions [11]. Previously, several studies [12,13] have been conducted around the globe to assess the preparedness and willingness of medical professionals in IMEs, and there is a paucity of research that has assessed the willingness and medical nature of IMEs in Saudi Arabia. This study identifies the willingness, medical nature, and challenges of physicians responding to an IME.

## Materials and methods

Study design

This cross-sectional study was carried out to investigate the nature and attitude of IME in Saudi Arabia. The data from the patients were collected through a structured online questionnaire.

Participants

The study included medical interns and postgraduate physicians. The study included participants working in Saudi Arabia. The target population consisted of 66,014 physicians, which led to a required sample size of 381, calculated via the Proportions Formula. Stratified convenience sampling ensured that the sample was representative of different regions, specialties, and levels of experience. Questionnaires were distributed through electronic medical communities from each major region.

Survey instrument and data collection

The survey was created using Google Forms in which the physicians were asked about their confidence and preparedness toward IMEs and the various factors that hinder them from aiding in an IME. It also gathered demographic details, such as age, gender, years of experience, and professional rank. Two medical experts reviewed the questions for clarity and relevance. Reliability testing was conducted, and the survey achieved a Cronbach’s alpha score of at least 0.70, which ensured consistency. The questionnaire was adapted from AlShamlan et al. after seeking their permission [14]. The questionnaire consisted of 14 questions about willingness to respond to IME and challenges encountered. Distribution was carried out through emails, professional networks, and social media platforms to reach physicians nationwide. Responses were measured on a five-point scale ranging from 1 (strongly disagree) to 5 (strongly agree), with scores compiled for analysis.

Data analysis

Data were analyzed using IBM SPSS Statistics for Windows, version 20.0 (IBM Corp., Armonk, NY). Quantitative data were expressed as mean and standard deviation. Qualitative data were expressed as numbers and percentages. Inferential statistics, including one-way ANOVA and independent t-tests, were performed to examine differences between groups based on factors such as age, gender, experience, and professional rank. A p-value of less than 0.05 was deemed significant.

Ethical considerations

Each participant provided informed consent before starting the survey. Prior to the data collection, approval was obtained from the Institutional Review Board (IRB) of Dar Al Uloom University (HP011352519). The data were collected in line with the ethical principles outlined in the Helsinki Declaration. The data were anonymized to ensure that there was no risk of bias. Data confidentiality was also maintained during the study.

## Results

A total of 368 participants were included in the study. The majority of participants were aged 30-39 years (n = 156, 42.4%), with a notable proportion aged 40-49 years (n = 145, 39.4%). Most had six to nine years of experience (n = 212, 57.6%) and held a resident qualification (n = 143, 38.9%), followed by general practitioners (n = 96, 26.1%). Regarding life support courses, 126 participants (34.2%) had basic life support (BLS), and 50 (13.6%) had advanced cardiovascular life support (ACLS). Travel frequency showed that more than half (n = 203, 55.2%) traveled more than three times a year, while 228 participants (62.0%) had attended or provided help in an IME. A similar proportion (n = 247, 67.1%) had encountered an IME before. There was a slightly higher proportion of males with BLS certification (n = 72, 35.5%, compared to n = 54, 32.7% in females), while more females reported having 10-15 years of experience (n = 34, 20.6% vs. n = 29, 14.3% in males). A higher percentage of males (n = 137, 67.5%) had attended or helped in IMEs compared to females (n = 91, 55.2%) (Table 1).

**Table 1:** Demographic details of the included participants

Variable		Male		Female		Overall	
		N	%	N	%	N	%
Age (years)	<30	33	16.3	22	13.3	55	14.9
	30-39	93	45.8	63	38.2	156	42.4
	40-49	70	34.5	75	45.5	145	39.4
	50-59	5	2.5	5	3.0	10	2.7
	≥60	2	1.0	0	0.0	2	0.5
Years of experience	<5	49	24.1	36	21.8	85	23.1
	6-9	119	58.6	93	56.4	212	57.6
	10-15	29	14.3	34	20.6	63	17.1
	>15	6	3.0	2	1.2	8	2.2
Professional qualification	Medical intern	29	14.3	20	12.1	49	13.3
	Resident	79	38.9	64	38.8	143	38.9
	General practitioner	45	22.2	51	30.9	96	26.1
	Specialist	29	14.3	19	11.5	48	13.0
	Consultant	21	10.3	11	6.7	32	8.7
Do you have basic life support (BLS)?	Yes	72	35.5	54	32.7	126	34.2
	No	131	64.5	111	67.3	242	65.8
Do you have advanced cardiovascular life support (ACLS)?	Yes	34	16.7	16	9.7	50	13.6
	No	169	83.3	149	90.3	318	86.4
What is your travel frequency?	More than 3 times per year	110	54.2	93	56.4	203	55.2
	Monthly	35	17.2	30	18.2	65	17.7
	Less than 1 per year	12	5.9	10	6.1	22	6.0
	2-3 times per year	46	22.7	32	19.4	78	21.2
Have you ever encountered an inflight medical emergency before?	Yes	136	67.0	111	67.3	247	67.1
	No	67	33.0	54	32.7	121	32.9
Attended or provided help in inflight medical emergencies?	Yes	137	67.5	91	55.2	228	62.0
	No	66	32.5	74	44.8	140	38.0

**Figure 1:**
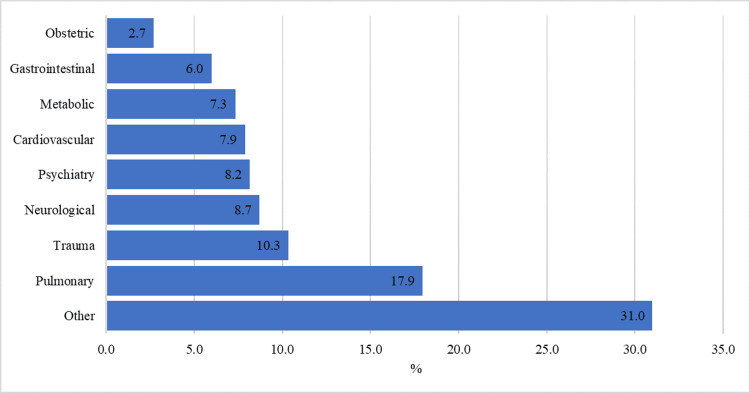
Nature of IMEs encountered by the physicians IME, inflight medical emergency

Figure 1 shows the nature of different IMEs. Pulmonary conditions were most common (n = 66, 17.9%), followed by trauma-related cases (n = 38, 10.3%). Neurological issues made up 32 (8.7%) cases, while psychiatric, cardiovascular, metabolic, and gastrointestinal conditions represented 30 (8.2%), 29 (7.9%), 27 (7.3%), and 22 (6.0%) cases, respectively. Obstetrics and gynecology cases contributed the smallest percentage at 10 (2.7%) cases. While the other (n = 113, 31%) group had major representation, it was determined by the respondent to list his encounter as other if not provided in the previous examples.

Table 2 summarizes physicians’ perspectives on volunteering in IMEs. A majority (64.2%, n = 236) agree or strongly agree that they believe physicians should volunteer, with 178 (48.4%) agreeing and 58 (15.8%) strongly agreeing. Motivation to volunteer is relatively high, with 247 (67.2%) agreeing or strongly agreeing, while 129 (35.1%) strongly agree and 118 (32.1%) agree. Barriers to volunteering are acknowledged by 230 (62.5%), including 79 (21.5%) strongly agreeing and 151 (41.0%) agreeing. While 226 (61.4%) feel prepared to respond to emergencies, concerns about legal issues are evident, with 225 (61.1%) agreeing or strongly agreeing that such worries deter them. Access to additional training and airline awareness campaigns are seen as key facilitators, with 249 (67.6%) and 245 (66.6%), respectively, agreeing or strongly agreeing. Many (n = 235, 64.1%) believe volunteering enhances professional skills, with 140 (38.0%) agreeing and 96 (26.1%) strongly agreeing. While 221 (60.1%) agree or strongly agree that airline staff are well-trained for such scenarios, comfort in making high-pressure decisions is slightly lower, with 200 (54.4%) agreeing or strongly agreeing (n = 124, 33.7% agreeing and n = 76, 20.7% strongly agreeing).

**Table 2:** Physicians’ perspectives on volunteering in inflight medical emergencies

Statement	Strongly agree	Agree	Neutral	Disagree	Strongly disagree
I believe that physicians should volunteer to assist in an inflight medical emergency.	58 (15.8%)	178 (48.4%)	125 (34.0%)	7 (1.9%)	0 (0.0%)
I’m motivated to volunteer in an inflight medical emergency.	129 (35.1%)	118 (32.1%)	99 (26.9%)	19 (5.2%)	3 (0.8%)
I face barriers when considering volunteering in an inflight medical emergency.	79 (21.5%)	151 (41.0%)	106 (28.8%)	25 (6.8%)	7 (1.9%)
I feel adequately prepared to respond to an inflight medical emergency.	86 (23.4%)	140 (38.0%)	107 (29.1%)	34 (9.2%)	1 (0.3%)
I believe that legal concerns prevent me from volunteering in an inflight medical emergency.	78 (21.2%)	147 (39.9%)	107 (29.1%)	29 (7.9%)	7 (1.9%)
I would be more likely to volunteer if I had access to additional training/resources.	108 (29.3%)	141 (38.3%)	97 (26.4%)	18 (4.9%)	4 (1.1%)
I think more awareness from airline companies would encourage physicians to volunteer.	92 (25.0%)	153 (41.6%)	102 (27.7%)	21 (5.7%)	0 (0.0%)
I believe volunteering in an inflight medical emergency can enhance my professional skills.	96 (26.1%)	140 (38.0%)	113 (30.7%)	18 (4.9%)	1 (0.3%)
I believe that airline staff are well-trained to handle inflight medical emergencies.	89 (24.2%)	132 (35.9%)	111 (30.2%)	32 (8.7%)	4 (1.1%)
I feel comfortable making medical decisions in high-pressure environments (e.g., airplanes).	76 (20.7%)	124 (33.7%)	114 (31.0%)	43 (11.7%)	11 (3.0%)
I would participate in training programs specifically for inflight medical emergencies.	89 (24.2%)	146 (39.7%)	102 (27.7%)	28 (7.6%)	3 (0.8%)
I worry that my involvement might lead to negative legal consequences.	75 (20.4%)	145 (39.4%)	113 (30.7%)	32 (8.7%)	3 (0.8%)
I believe that my lack of specific training for inflight emergencies limits my willingness to volunteer.	88 (23.9%)	139 (37.8%)	97 (26.4%)	41 (11.1%)	3 (0.8%)
I feel a moral obligation to assist in a medical emergency regardless of the setting.	94 (25.5%)	141 (38.3%)	112 (30.4%)	20 (5.4%)	1 (0.3%)

The study found significant differences in attitudes toward volunteering in IMEs based on several factors. Participants with BLS training had lower mean scores compared to those without BLS (p < 0.001), while those who had encountered an IME or provided help reported higher scores (p < 0.001). Age also influenced attitudes, with younger participants (under 30 years) having the highest scores and those over 60 years having the lowest scores (p < 0.01). Experience levels and professional rank also showed significant differences, with those having less experience (less than five years) and higher-ranking professionals (consultants and specialists) displaying more positive attitudes (p < 0.01). However, no significant differences were found between gender, ACLS training, or travel frequency categories (Table 3).

**Table 3:** Relationship between physicians’ attitudes and various study variables

Variable		Mean ± SD	p-value
Gender	Male	53.39 ± 8.07	0.176
	Female	52.26 ± 7.85	
Do you have basic life support (BLS)?	Yes	50.64 ± 7.11	<0.001
	No	54.05 ± 8.17	
Do you have advanced cardiovascular life support (ACLS)?	Yes	52.50 ± 6.30	0.714
	No	52.95 ± 8.22	
Have you encountered an inflight medical emergency before?	Yes	54.64 ± 7.81	<0.001
	No	49.30 ± 7.10	
Have you attended or provided help in an inflight medical emergency before?	Yes	54.88 ± 7.78	<0.001
	No	49.64 ± 7.23	
Age (years)	<30	54.8545 ± 7.03	<0.01
	30-39	54.5897 ± 7.54	
	40-49	50.5862 ± 8.26	
	50-59	50.1000 ± 6.77	
	>60	46.5000 ± 4.94	
Experience (years)	<5	54.6941 ± 7.16	0.039
	6-9	52.7736 ± 8.16	
	10-15	50.9048 ± 8.23	
	>15	52.2500 ± 5.75	
Professional rank	Medical intern	54.5306 ± 6.81	<0.01
	Resident	50.1259 ± 7.87	
	General practitioner	53.5729 ± 7.80	
	Specialist	55.2292 ± 8.47	
	Consultant	57.1250 ± 5.88	
Travel frequency	More than 3 times/year	53.4433 ± 7.85	0.015
	Monthly	50.0154 ± 8.26	
	Less than 1 per year	53.1818 ± 4.85	
	2-3 times/year	53.7436 ± 8.37	

## Discussion

This study was conducted to investigate the experiences and challenges physicians face when responding to IMEs in Saudi Arabia. The findings of the study showed that the majority of the participants had a positive attitude toward handling an IME. Furthermore, the majority of the participants were not reluctant to participate in an IME, as out of 67.1% of participants who encountered an IME, 62% participated. Our findings align with the study by AlShamlan et al., who reported that approximately one-third of the respondents encountered an IME, and the majority of those participated in it [14]. Similarly, another study by Ng and Abdullah reported that 69.2% of their study participants showed their willingness to participate in an IME; however, if anyone else was involved in providing medical support, they became reluctant [15].

The study also showed a predominance of respiratory conditions and trauma and allergy-related cases among the specific medical emergencies encountered. Similar to our study, Peterson et al., in their study, which included flight records of 11,920 IMEs, reported that syncope or presyncope was the most common IME, followed by respiratory complication observed in 12.1%. In almost half of these IMEs, physicians intervened. Furthermore, 7.3% of IMEs led to aircraft diversion [16]. In the present study, cardiovascular IMEs were only reported by 7.9% compared to 25% by AlShamlan et al. [14]. They also noted that 19% of the IMEs were caused by a pulmonary condition. Hinkelbein et al. reported that 40.0% of cases had a cardiovascular diagnosis, followed by neurological disorders (17.8%) [17]. In this study, 8.2% of individuals experienced psychiatric symptoms. These symptoms can vary widely during air travel and can range from mild anxiety to severe episodes like acute psychosis [18]. The confined spaces in the flight can further exacerbate these situations. Verbal reassurance is generally sufficient to calm the patients in such scenarios [19].

Many participants highlighted the lack of specialized training as a major obstacle. Alarifi and ALRowais also found a strong need for additional training, with 93.2% of respondents emphasizing its importance [20]. This highlights the urgent need to better prepare medical professionals for IMEs and calls for medical training programs to address this gap effectively. Their study revealed that nearly all participants (97.9%) held life support credentials, such as BLS and ACLS [20]. This sharply contrasts with our study, where only 34.2% reported having BLS certification, and just 13.6% had ACLS. The current study also showed that participants who had BLS had significantly low mean attitudes compared to those who did not. This seems like an anomaly in our results, which can be explained by the fact that only a small number of participants in our study had BLS. Proper medical training is crucial because it equips individuals with the essential knowledge and skills to handle emergencies effectively, whether on the ground or during inflight situations [6].

Several studies have been conducted regarding IME training with positive outcomes. A simulation program at Vanderbilt University Medical Center, Nashville, TN, trained participants on roles, resources, and patient care during IMEs, which they found highly effective for boosting preparedness [21]. Ohio State University Wexner Medical Center, Columbus, OH, used a grounded airplane for realistic simulations of medical scenarios, such as anaphylaxis and pulmonary embolism, with discussions on topics such as aeromedical considerations. Participants reported increased knowledge of ACLS protocols and felt more confident in their skills after the training [22]. Medico-legal concerns are a significant barrier influencing whether or not to participate in an IME. Alarifi and ALRowais noted that 57.7% of respondents expressed concerns about medico-legal issues [20]. Similarly, in our study, 20.4% strongly agreed, and 39.4% agreed that legal implications contributed to their reluctance to participate in IMEs. Fear of litigation or professional consequences often dissuades physicians from intervening, even when they are willing and capable.

In the United States, the Aviation Medical Assistance Act, often called the “Good Samaritan” shield, protects passengers who step in to provide medical help during a flight [6]. This law shields them from being held legally responsible unless their actions involve gross negligence or intentional harm. When it comes to flights outside the United States, the rules are more complex. Unlike in the United States, many other countries do not offer the same legal protection for volunteers providing medical assistance, which highlights the need for policymakers to issue a similar law and to make it known to encourage volunteers with medical backgrounds. Additionally, the expectation to help varies by country. In most cases, off-duty medical professionals are not required to step in during inflight emergencies [23]. In the present study, the majority of the participants were residents or general practitioners. Peterson et al. reported that in almost half of the cases, IME was assisted by a physician and in 25% by flight crew [16]. It is important to know that the flight crew is given BLS training [24]. When there is more than one volunteer onboard, it is helpful for them to discuss their skills and experience. For instance, a physician who specializes in a non-related field might not be the best fit to manage a particular situation compared to someone with more relevant emergency training [6].

Additionally, the high-pressure environment in an aircraft can exacerbate stress levels. Only 54.4% of participants felt comfortable making critical decisions in such settings. The constrained space, lack of familiarity with the available medical kit, and lack of support from other medical staff add to the challenges [25]. Several significant differences between the participant groups were observed in the present study. Participants who had encountered or aided in an IME before had better attitudes compared to those who did not. This finding is in line with AlShamlan et al. [14]. However, no difference in attitude was observed between males and females. These findings contrast with those of AlShamlan et al., who found higher willingness in male participants regarding participation in IME [14]. In the present study, no significant difference was observed in attitude based on the experience of the participants. However, Sharaf et al. reported that healthcare workers with more than 15 years of experience were more willing to participate in IME [26].

There are several limitations in this study that could have influenced the results. Firstly, the retrospective nature of the study can add bias to the findings, like recall bias, which was unavoidable in the present case. Secondly, the study included a relatively small sample size, which could have influenced the results, as evidenced by only a small number of participants reporting BLS, which is a key finding that needs to be evaluated further. Another limitation is the potential underrepresentation of certain demographic groups, such as older physicians or those less familiar with digital platforms, as the survey was distributed online. Furthermore, the study did not account for cultural or organizational factors specific to the settings of IMEs that might influence physicians’ attitudes or willingness to volunteer.

## Conclusions

In conclusion, the findings of the present study show that the majority of the participants have positive attitudes and are willing to participate in an IME. Participants with prior emergency experience, younger participants, and those with less experience had more positive attitudes regarding aiding in IME. Respiratory issues are the most commonly encountered IME by physicians. Despite the positive attitude of the physicians, barriers such as medico-legal issues and lack of proper medical training can influence the decision to participate in an IME.
